# Changes in Mechanical Properties of Medium Manganese Steel After Forming, Press Hardening, and Heat Treatment

**DOI:** 10.3390/ma18061196

**Published:** 2025-03-07

**Authors:** Radek Leták, Ludmila Kučerová, Hana Jirková, Štěpán Jeníček, Filip Votava

**Affiliations:** 1 Regional Technological Institute, University of West Bohemia, Univerzitní 8, 301 00 Pilsen, Czech Republic; skal@fst.zcu.cz (L.K.); jeniceks@fst.zcu.cz (Š.J.); votavaf@fst.zcu.cz (F.V.); 2 Department of Materials and Engineering Metallurgy, University of West Bohemia, Univerzitní 8, 301 00 Pilsen, Czech Republic; hstankov@fst.zcu.cz

**Keywords:** medium manganese steels, intercritical annealing, press hardening, high-strength steels

## Abstract

Solutions and new processes are continually being developed to produce components demonstrating high strength and elongation. This paper focuses on medium manganese steel with a composition of 0.2% carbon, 3% manganese, and 2.15% aluminium (by weight percent). The mechanical properties of the steel and the effect of aluminium and manganese on the microstructure are investigated. The steel sheets are shaped into omega profiles using a press tool, followed by the intercritical annealing of the samples to enhance their ductility. Before the experiment, the anticipated values were a tensile strength (UTS) of approximately 1100 MPa and elongation within 30–35%. A key objective was to achieve a microstructure that incorporates residual austenite. The experimental parameters were carefully derived from an extensive exploration to identify potential weaknesses in the experiment. The main parameters selected were the intercritical annealing (IA) temperature and IA dwell time. The results revealed that the highest recorded UTS was 1262 ± 6 MPa, while the maximum elongation achieved was 16 ± 1%.

## 1. Introduction

In most of the world, the automotive industry is one of the primary driving forces for developing and advancing technologies and materials. It is also an important asset that keeps the economies of many countries running. Automotive research is also heavily concerned with an ecological view of production and subsequent sustainability [1]. One of the primary goals is to minimise the weight of vehicles. The lighter the car body, the lower the fuel consumption and the lower the carbon footprint. In this way, the environmental standards set by the European Union are also met [2,3]. The most important thing, however, remains the protection of the passengers, and only steels with high mechanical values will ensure this [4]. In addition to weight reduction to eliminate harmful exhaust fumes, there is a strong emphasis on design, high manufacturing efficiency, and the ease of assembly of individual parts [5,6]. Moreover, all of these areas are constrained by the system’s financial policy, where cost savings are significant and essential [7,8].

The development of high-strength steels, which are valued for their high strength, good formability, and excellent elongation, is constantly accelerating. These steels play a significant role in ensuring passenger safety, particularly in specific body segments [9].

Car bodies are mechanically tested and the results are strictly evaluated. These tests are essential for passenger safety. One specific body segment is the B-pillar. In many cases, B-pillars are made of a single material with different mechanical properties in other places. This difference in properties is significant for the safety of the car. The upper part of the pillar should have better strength and less ductility, while the lower part should have lower strength and higher ductility to ensure the safety of the occupants in the event of a crash [10].

Press-hardening technology facilitates the processing of ultra-high-strength hardenable sheets specifically designed for heat treatment. The primary material employed in this technology is 22MnB5-based steel, which has been developed specifically for this application. Çavuşoğlu et al. studied manganese-boron steels with a strength of approximately 550 MPa [11].

Third-generation steels, such as medium manganese steels (MMnSs), are frequently utilised due to their enhanced mechanical properties compared to first-generation steels. Research on medium manganese steels indicates a manganese content ranging from 3% to 12% [11]. At present, the heat treatment of steels with medium manganese content is carried out by the austenite conversion process. Ye et al. investigated the microstructure of medium manganese steel, characterised by either ultrafine-grained ferrite combined with metastable austenite or martensite derived from austenite transformation. Carbon and manganese are crucial in stabilising austenite as it forms from ferrite. The resulting microstructure consistently exhibited a duplex or triplex phase configuration [12].

A significant quantity of austenite with adequate stability is the main factor that controls the properties of medium manganese steels. To increase the austenite content and improve its stability, Wang et al. and Hu et al. prepared a mixed morphology of lath-like and spherical grains with a wide size distribution by using a heterogeneous structural design and the hot annealing of medium manganese steel [13,14].

Many factors influence the final state of the microstructure and, therefore, the mechanical properties of the sample. The most important ones are the grain size, the temperature dwell time at IA, and the rate of hot deformation. Kotrba et al. studied the impact of the grain size on the mechanical properties of medium manganese steel. Their findings revealed that an elevation in the austenitisation temperature increased the grain size and martensitic lath size within the final predominantly martensitic microstructure [15,16,17]. Other critical factors include the IA temperature and the holding time at IA. Cao et al. and Mohapatra et al. have demonstrated that the temperature should not be close to Ac1 or Ac3. At low temperatures, insufficient austenitisation of the material occurs, whereas, at high temperatures, grain coarsening takes place [18,19]. Hot deformation also affected the microstructure when Krbaťa et al. evaluated the influence of hot work parameters such as the deformation rate on the flow curve and material parameters [20].

Several techniques are known to achieve various microstructures and, thus, different mechanical properties in an individual piece. An example is the work of Jirková et al., where induction heating was used and applied only to specific areas [21,22,23,24]. Which approach leads to the best combination of the required ductility and strength of the material?

This paper deals with intercritical thermal annealing and its impact on the material. It is essential to compare the ductility after the second step, i.e., after intercritical annealing, when it is expected to be around 30%. It should be higher than when using press hardening.

Another aim of the paper is to answer how the experimental steel responds to IA. The experimental steel was designed with an aluminium addition of 2.15%. The microstructure is examined and compared to other works where aluminium is not present in such an amount. No additional percentage of aluminium was added to prevent carbides κ from forming [14,25].

This process is not definitive, and it will be followed up with controlled multiple heating and cooling processes using induction annealing technologies. This paper aims to describe the effect of heat treatment after the forming process on increasing the ductility and maintaining a high ultimate strength.

## 2. Materials and Methods

The experimental material selected for this study was medium manganese steel. Its chemical composition included 0.21% carbon, with manganese (3.06%) and aluminium (2.15%) as the primary alloying elements. Manganese, together with carbon, can stabilise austenite. Aluminium makes the reverse austenitic transformation faster. Aluminium reduces the stability of cementite and thus prevents the formation of carbides. It promotes its decomposition, facilitating carbon diffusion into ferrite and accelerating the reverse transformation to austenite. It influences the Ac1 temperature (the onset of austenitic transformation during heating), which may slightly increase, while simultaneously accelerating the transformation kinetics. The presence of aluminium can affect the distribution of austenite in the microstructure, which is crucial for the resulting mechanical properties. It promotes a more homogeneous distribution of austenite and can contribute to improved mechanical properties, such as increased toughness and enhanced deformation behaviour [26,27,28]. Silicon in the experimental steel slows down the rate of carbide precipitation in bainite, which promotes the stabilisation of residual austenite (RA). Due to its chemical composition, this steel can form a functional component with a multiphase structure containing RA in the matrix. The material used is classified as third-generation high-strength steel. The desired mechanical properties are achieved in the final product using the TRIP effect. The chemical composition is given in Table 1. The experimental steel was cast into a cylindrical ingot. The weight of the ingot was 168 kg. Then, a 4.2 mm thick sheet was fabricated. Cold rolling was carried out in 16 steps with a reduction in size of 5%. The final thickness of the sheet was 1.8 mm. Specimens of 120 × 80 mm were cut from the sheets by waterjet. Then, homogenisation annealing in a protective argon atmosphere at 680 °C with a hold time of 2 h was used to create a ferritic structure with globular carbides.

**Figure 1:**
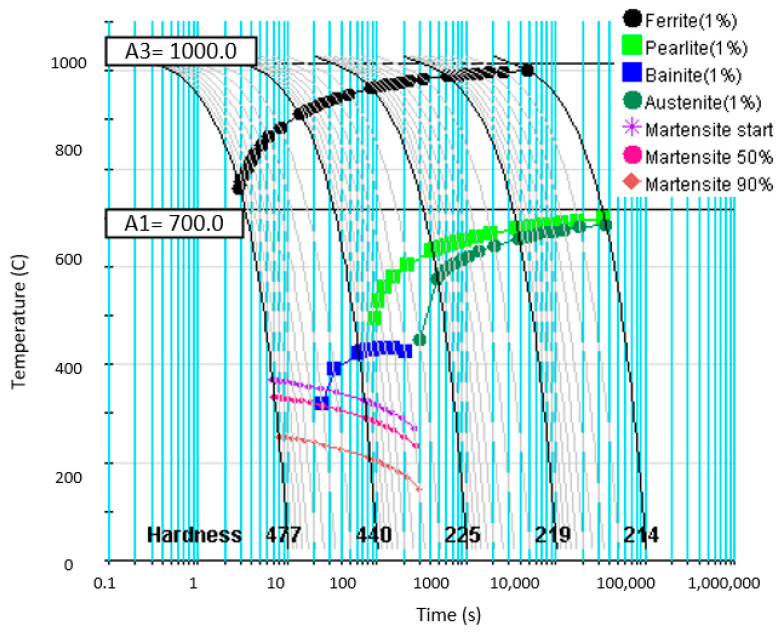
CCT Diagram showing temperatures A1 and A3.

The phase transformation temperatures and the Ac_1_ and Ac_3_ temperatures were determined by calculation in JmatPro 12.1 software. All of the temperatures were generated under very similar conditions. Experimental values were derived based on the work by Kozlowski et al. and the work of Wu [29,30]. The critical cooling rate was determined to be 100 °C/s. The cooling time at this temperature was 10 s. The grain size was determined to be 9 ASTM. The onset temperatures of the phase transformations are noted in Figure 1.

### 2.1. Press Hardening

The press hardening was carried out in a hydraulic press type CKW s6000, with an omega profile tool. The profile depth of the tool was 30 mm. Processing started by heating the sample in a furnace without a protective atmosphere to a temperature of 1000 °C and holding it at that temperature for 30 min. Then, the experimental steel sheet was transferred to the press tool at room temperature (RT). Removing the specimen from the furnace and transferring it to the prepared tool took 2 s. This was followed by closing the tool and performing the pressing operation. Closing the tool resulted in the formation of an omega profile. After the tool closure, a tool dwell of 1 or 5 s was performed. The residence time of the specimen in the jaws was controlled by a timer on the machine.

### 2.2. Intercritical Annealing of Omega Profiles

The samples were subjected to intercritical annealing to increase their elongation values. The temperatures for the intercritical annealing were chosen in the following intervals of 50 °C: 700, 750, and 800 °C. The holding time was 30 min. All of the temperatures were selected in the region between Ac1 and Ac_3_. The temperature values were obtained using JmatPro V12.1. This part of the experiment aimed to create a structure containing residual austenite and martensite.

The reason for making this microstructure is because of its theoretically higher ductility than a microstructure consisting of only martensite [12].

### 2.3. Evaluation Methods

The microstructure evaluation was performed identically for all of the samples. An Olympus light microscope evaluated the microstructure from a cross-section (Olympus, Tokyo, Japan). The detailed microstructure was assessed using a Tescan scanning electron microscope (Tescan, Brno, Czech Republic). The hardness was also measured on the samples, averaging five impressions, using the Vickers method; the load was 10 kP (HV10). The time for one impression was 10 s. Four impressions were always taken and then averaged (Wolpert Wilson Instruments, 432-SVD). A Discotom 6 (Discotom, Paris, France) metallographic saw was used to cut the samples. A Citopress single-chamber press and Labopol 21 metallographic grinders and polishers were used to prepare the samples (Labopol 21, Paris, France; Citopress, Paris, France). The Vilella–Bain solution was used to etch the structures for the observation.

The chemical composition was determined from three measurements using a Tasman Q4 optical Emission Spectrometer (Tasman Q4, Rudice, Czech Republic).

**Figure 2:**
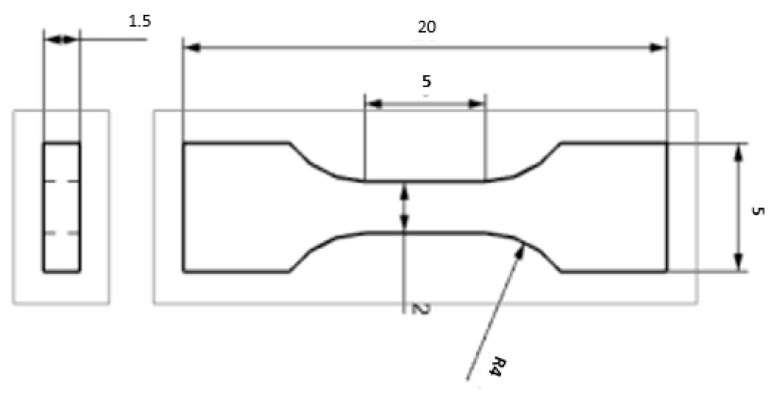
A mini-tensile test specimen.

**Figure 3:**
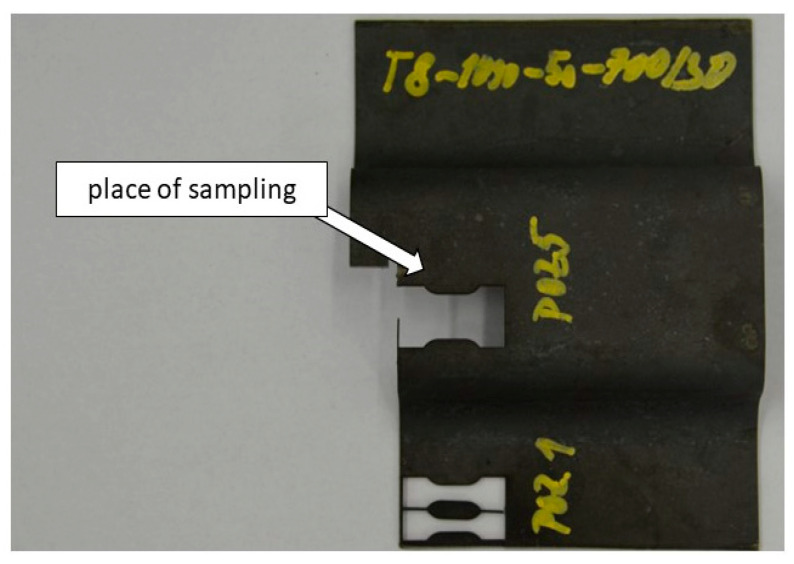
Omega profile with tensile test samples.

The tensile testing was conducted according to EN ISO 6892-1 method A on a ZWICK 250 universal machine with a maximum force of 250 kN (ZWICK 250, Ulm, Germany). Two samples were tested for each parameter variant. The length of the active part was 5 mm. The cross-section was 2 × 1.5 mm. The geometry of the test body is presented in Figure 2 and Figure 3.

## 3. Results

**Figure 4:**
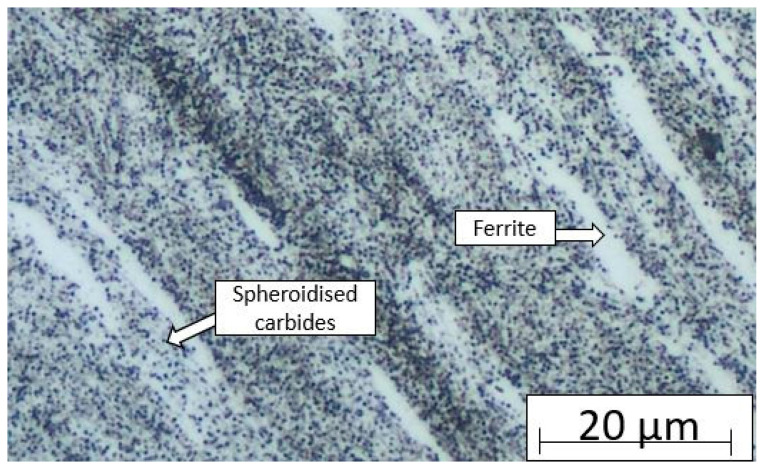
Microstructure after cold rolling and annealing.

The metallography of the material before heat treatment and press hardening was analysed after cold rolling and annealing at 680 °C for 2 h. The microstructure comprised a ferritic matrix and spheroidised carbides (Figure 4). The hardness value was determined to be HV10 354 ± 2.

**Figure 5:**
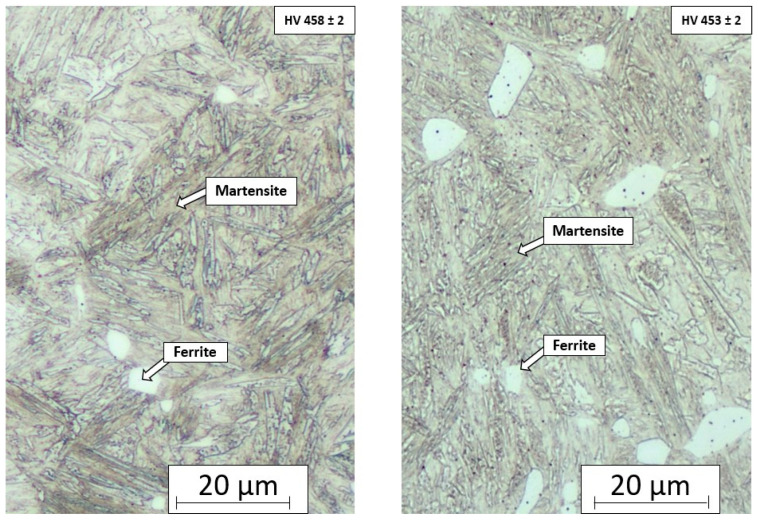
Microstructure showing the martensitic structure and ferrite areas after quenching in the tool for 1 s (**left**) and for 5 s (**right**).

The first step was press hardening the sample in the tool, which was quenched for 1 s. After processing with a quenching time of 1 s, the microstructure was a mixture of martensite and ferrite grains (see Figure 5). The ultimate strength was around 1326 ± 15 MPa, and the ductility was 19 ± 1.1. The hardness was 459 ± 2 HV10. The values are recorded in Table 2. When the quenching time in the tool was extended to 5 s, the structure showed visible changes but was still composed of a combination of martensite and ferrite islands. The mechanical properties were also similar. The ultimate strength was 1392 ± 35, the ductility was 18.3 ± 0.2, and the hardness was around 453 ± 5 HV10. The values were identical, but samples pressed for 5 s were used for the next step because of their slightly higher ultimate strength. The material’s mechanical properties after the press-hardening process are recorded in Table 2. In this step, in addition to the mechanical properties, obtaining a microstructure in which martensite predominates was important. Therefore, the variant with quenching in the tool for 5 s was chosen. Martensite transitions more quickly to the austenitising temperature, forming a homogeneous austenitising microstructure. This reduction in time is essential in industry. Heating to IA temperature will take less time here [31].

After pressing, the sample was in contact with the press tools only for cooling purposes. Almost no pressure was applied to the surfaces. Figure 5 shows the tensile testing of samples after press hardening.

### Intercritical Annealing (IA)

After the annealing process at 700 °C, a strongly tempered martensitic structure with ferrite islands was obtained. Because 700 °C is exactly the Ac1 temperature of this steel, no transformation of the crystal lattice or austenite formation occurred at this temperature. Only the structure was tempered thereafter, and the hardness value decreased from the original 458 HV10 to 273 HV10. The mechanical properties are given in Table 3.

For the sample annealed at 750 °C, the structure was a mixture of strongly tempered martensite with ferritic grains. Wang et al. described a similar structure with tempered martensite and secondary martensite in their paper. However, there were no ferritic grains in their structure because multiphase annealing was used [13]. The hardness values decreased similarly at 700 °C to 264 HV10. This was due to the even further tempering of the original martensitic microstructure formed during tool forming. The same effect was found at the highest annealing temperature of 800 °C when the hardness dropped to 233 HV10.

IA led to an increase in the hardness value and an increase in the ultimate strength compared to the base material. Thus, when compared with the process after press hardening, the mechanical property values did not come out better. Specifically, at 700 °C, the ultimate strength was 1037 MPa and the ductility value was 16%. At an annealing temperature of 750 °C, the ultimate strength was 1163 MPa and, at an IA temperature of 800 °C, the ultimate strength was 1226 MPa. The increase in strength compared to the base material with increasing annealing temperature can be explained by the formation of austenite that occurs when heating to higher annealing temperatures. However, this austenite was unstable enough and transformed into martensite when cooling to RT. This structure was considerably strained after the IA process, so the ultimate strength was lower than when the sample was cooled in the tool. Neither of these phenomena had a positive effect on the ductility values.

**Figure 6:**
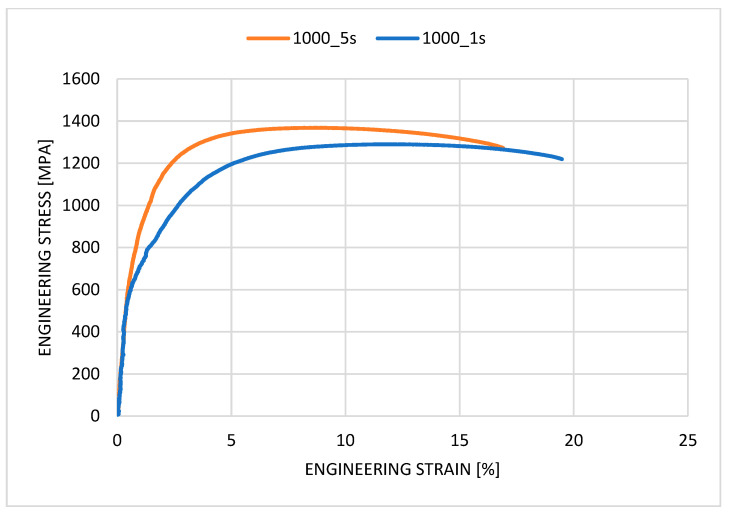
Tensile test results of the samples after press hardening.

**Figure 7:**
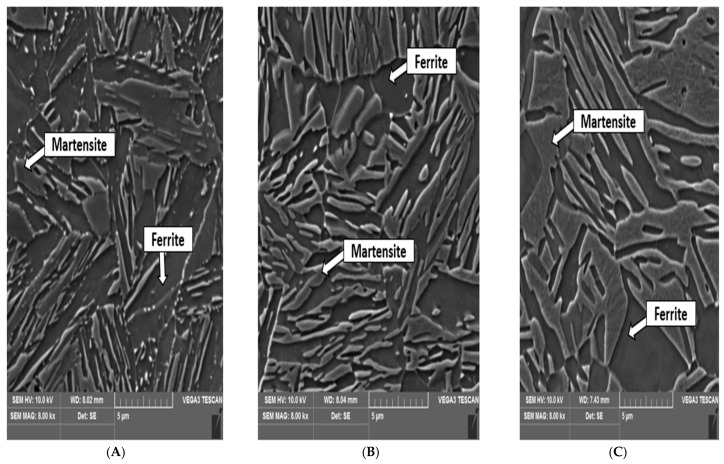
(**A**) The temperature tested at 700 °C; (**B**) the temperature tested at 750 °C; (**C**) the temperature tested at 800 °C.

Figure 6 shows the microstructures after the heat treatment. Figure 7A shows the microstructure after IA at 700 °C. The microstructure shows no areas with carbides, such as cementite. There are only areas with martensite and ferrite. A microstructure with a bainitic morphology consisting of a combination of ferrite and martensitic laths was observed. This type of microstructure was found in all samples regardless of the IA temperature. Figure 7B again shows the ferritic–martensitic structure. The last microstructure image, shown in Figure 7C, is also ferritic–martensitic, but there is already a noticeable difference. This image is after IA at 800 °C, and the martensitic laths and islands are much larger than the martensitic areas shown in Figure 7A, where the annealing temperature was 700 °C. None of the three microstructures contained any carbides, as was the case for Xu et al. [32]. In our case, the carbides dissolved and no longer formed during cooling to RT. Their absence may have been aided by the aluminium added to the structure for this purpose. The microstructures also confirmed the mechanical properties, where the strength and hardness limit of the tested samples increased with increasing temperature. 

## 4. Discussion

Medium manganese steels similar to 22MnB5 steel were developed for use in the press-hardening process. The 22MnB5 steel was selected for comparison with the experimental steel. Before the actual hardening process in the tool, the strength limit of the 22MnB5 steel was around 550 MPa. However, the ductility was around 5% [11,33].

The first step was press hardening, where, after quenching in the tool for 5 s, the experimental steel had a strength limit of 1392 MPa with a ductility value of approximately 18%. The result after the first part of the experiment was in agreement with other research studies by Costa et al. and Zhou et al. The structure showed a multiphase microstructure consisting of martensitic regions with ferritic grains. Manganese steels can achieve a multiphase microstructure consisting of a martensitic region that contains small ferritic islands [34,35]. The reason for ferrite formation after hardening is the short residence time in the jaws. In the investigations of Grajcar et al., hardening was performed on steel with 3% Mn, and ferrite did not appear in the structure due to the longer cooling time [36]. The results from testing the samples after intercritical annealing show that, as the heating temperature increases, we also see an increase in the ultimate tensile strength compared to the base material.

Initial tests conducted at 700 °C showed a tensile strength of 1037 MPa, an elongation of 16%, and a hardness of 260 HV10. When the temperature increased to 750 °C, the tensile strength rose to 1163 MPa, the elongation remained stable at approximately 16.5%, and the hardness slightly decreased to 254 HV10. At the highest temperature of 800 °C, the tensile strength increased to 1262 MPa. However, the elongation reduced to 12.2% and the hardness rose to 356 HV10. These findings suggest a notable increase in the tensile strength with higher processing temperatures, though a reduction in the ductility accompanies this improvement.

Comparable results have been reported in other studies, such as the work by Wang et al., where tensile strength values of around 1100 MPa were observed, and the ductility was around 20%. These results reflect similar trends in the mechanical properties as the processing temperature increases. However, the material studied in this case had a higher manganese content. This difference in materials could have caused changes in the elongation [13].

The microstructure was predominantly composed of martensite with ferrite islands. The martensite formation occurred during the final slow cooling of the sample from the IA temperature. The austenite formed during IA was not sufficiently stable and subsequently transformed into martensite. The same has been discussed in the work by Zou and Yan and their teams [37,38]. It can be assumed that the main reason for this transformation was the short duration of the IA process, which did not allow the manganese to effectively stabilise the austenite [39]. Another factor contributing to austenite instability may be the absence of other supporting steps to stabilise the retained austenite, such as QP processing or a second IA step, as reported by Chandan et al. [40].

Furthermore, ferritic grains were observed within the microstructure. This phase has also been reported in the experiments conducted by Mou et al. and Kozlowska et al., whose research on medium manganese steels identified ferrites after the initial IA stage. In their studies, the material’s ductility following annealing was also lower, at approximately [18%] [30,41]—the high strength of the corresponding samples and reduced ductility support this microstructural evolution.

## 5. Conclusions

The tested samples were made of medium manganese steel and were processed using press-hardening technology. After processing, their mechanical properties were tested and compared with the results from the 22MnB5 steel.

1. After press hardening, a predominantly martensitic microstructure was achieved.
2. The ultimate strength of the processed material was approximately 1350 MPa, and the surface hardness of the samples reached 350 HV10.
3. The ductility of the material after press hardening was measured at 19%.
4. Intercritical annealing with heating between Ac1 and Ac3 resulted in a change in the microstructure but did not result in a noticeable shift in the ductility.
5. Of the annealed experimental steel samples, the sample annealed at 800 °C had the highest ultimate strength, reaching 1262 MPa, and the ductility of this particular sample was measured to be 12.2%.
6. In contrast, the sample annealed at 750 °C reached a ductility of more than 16%, but the strength was 1163 MPa.

The ultimate strength values were very good after intercritical annealing, but the ductility was well below expectations due to the lack of stabilised austenite. The formation of a structure having the mechanical properties corresponding to the ones in the introduction of the article needs to be achieved by further thermal processes.

## Figures and Tables

**Table 1:** Chemical composition of the experimental material (wt. %) determined by a Bruke Q4 Tasman optical spectrometer.

Chem. Comp.	C	Mn	Al	Si	Cr	Cu	Nb	Ni	P	S
wt. %	0.21	3.06	2.15	0.57	0.16	0.069	0.057	0.048	0.006	0.003

**Table 2:** Mechanical properties after press hardening.

Hardening Time in the Tool [s]	Ultimate Strength (R_m_) [MPa]	Elongation (A) [%]	Hardness HV10 [-]
1	1326 ± 15	19.1 ± 1.1	459 ± 2
5	1392 ± 35	18.3 ± 0.2	453 ± 5

**Table 3:** Mechanical properties after intercritical annealing.

Hardening Time in the Tool [s]	Annealing Temperature [°C]	Ultimate Strength (R_m_) [MPa]	Elongation (A) [%]	Hardness HV10 [-]
5	700	1037 ± 34	16 ± 1	260 ± 4
5	750	1163 ± 69	16.5 ± 3.5	254 ± 4
5	800	1262 ± 6	12.2 ± 0.4	356 ± 7

## Data Availability

The raw data are not publicly available due to ongoing research.
